# Retrospective analysis of outcomes for pediatric acute lymphoblastic leukemia in South American centers

**DOI:** 10.3389/fonc.2023.1254233

**Published:** 2023-10-30

**Authors:** Caitlyn Duffy, Dylan E. Graetz, Arturo M. Zapata Lopez, Angela K. Carrillo, Godwin Job, Yichen Chen, Meenakshi Devidas, Sandra Alarcón Leon, Sol Aponte Bonzi, Pedro Cardona Flores, Lizeth Escobar Torres, Eddy Hernández Broncano, Soledad Jiménez Jaramillo, Ma Ofelia Zelada, Romulo Reaño Novoa, Angelica Samudio, Gissela Sánchez-Fernandez, Erika Villanueva, Monika L. Metzger, Paola Friedrich, Sima Jeha

**Affiliations:** ^1^ St. Jude Children’s Research Hospital, Department of Global Pediatric Medicine, Memphis, TN, United States; ^2^ Departamento de Oncología Pediátrica, Instituto Nacional de Enfermedades Neoplasicas, Lima, Peru; ^3^ Departamento de Hemato Oncología Pediátrica, Hospital de Clínicas, Facultad de Ciencias Médicas, Universidad Nacional de Asunción, Asunción, Paraguay; ^4^ Hospital Santa Cruz Caja Petrolera Salud (CPS), Hemato-Oncología Pediátrica, Santa Cruz, Bolivia; ^5^ Hospital SOLCA Núcleo Quito, Hemato-Oncología Pediátrica, Quito, Ecuador; ^6^ La Sociedad de Lucha Contra el Cáncer (SOLCA) Núcleo de Loja, Oncohematóloga Pediatra, Loja, Ecuador; ^7^ Médecins Sans Frontières, Geneva, Switzerland

**Keywords:** pediatric, acute lymphoblastic leukemia, survival, low-and middle-income country, multinational, consensus-derived

## Abstract

**Introduction:**

Acute lymphoblastic Leukemia (ALL) is the most common pediatric malignancy. While the survival rate for childhood ALL exceeds 90% in high-income countries, the estimated survival in low-and middle-income countries ranges from 22-79%, depending on the region and local resources.

**Methods:**

This study retrospectively reviewed demographic, biological, and clinical parameters of children under 18 years of age with newly diagnosed ALL presenting between 2013-2017 across five pediatric centers in 4 countries in South America. Survival analyses were estimated using the Kaplan-Meier method.

**Results:**

Across the five centers, 752 patients were analyzed (Bolivia [N=9], Ecuador [N=221], Paraguay [N=197], Peru [N=325]) and 92.1% (n=690) patients were diagnosed with B-cell and 7.5% (n= 56) with T-cell ALL. The median age was 5.5 years old (IQR 7.29). At diagnosis, 47.8% of patients were categorized as standard and 51.9% as high risk per their institutional regimen. Advanced diagnostics availability varied between modalities. MRD was evaluated in 69.1% of patients; molecular testing was available for ETV6-RUNX, BCR-ABL1, TCF3-PBX1, and KMT2A-rearranged ALL in 75-81% of patients; however, karyotyping and evaluation for iAMP21 were only performed in 42-61% of patients. Central nervous system (CNS) involvement was evaluated at diagnosis in 57.3% (n=429) patients; of these, 93.7% (n=402) were CNS 1, 1.6% (n=7) were CNS 2, 0.7% (n=11) were CNS3, 1.9% (n=8) had cranial nerve palsy, and 2.1% (n=9) results unavailable. Chemotherapy delays >2 weeks were reported in 56.0% (n=421) patients during treatment. Delays were attributed to infection in 63.2% (n=265), drug-related toxicities in 47.3% (n=198), and resource constraints, including lack of bed availability in 23.2% (n=97) of patients. The 3-year Abandonment-sensitive EFS and OS were 61.0±1.9% and 67.2±1.8%, respectively. The 3-year EFS and OS were 71.0±1.8% and 79.6±1.7%, respectively.

**Discussion:**

This work reveals opportunities to improve survival, including addressing severe infections, treatment interruptions, and modifications due to drug shortages. In 2018, healthcare professionals across South America established the Pediatric Oncology Latin America (POLA) group in collaboration with St. Jude Children’s Research Hospital. POLA collaborators developed an evidence-based, consensus-derived, adapted treatment guideline, informed by preliminary results of this evaluation, to serve as the new standard of care for pediatric ALL in participating institutions.

## Introduction

Acute lymphoblastic Leukemia (ALL) is the most common pediatric malignancy worldwide. While the overall survival for ALL in high-income countries (HIC) exceeds 90% (1–3), the estimated survival in low-and middle-income countries (LMIC) widely varies from 22-79% (4). In 2007, a global analysis of 68 countries demonstrated the highest incidence of ALL in Hispanic populations (5). Subsequent studies showed a more rapid increase in ALL incidence over time and a comparatively poorer prognosis in these children (6). The Children’s Oncology Group (COG) recently reported that Hispanic children with B-cell ALL had a lower 5-year event-free survival (EFS) and overall survival (OS) compared to non-Hispanic white children (83.6% vs. 88.3%, P<0.0001, 90.4% vs 94.1%, P<0.0001 respectively) (7). Historically, this survival disparity has been attributed to biological etiologies such as relative differences in tumor biology, pharmacogenomics, and comorbidities, as well as socioeconomic differences, including limited access to clinical trials, structural barriers to care, inconsistent access to standard diagnostics tests and essential medications, delay in diagnosis and therapy initiation, time spent with providers, education received, and adherence (8–10). The COG recently found that while survival disparities are attenuated when adjustments for specific biologic prognosticators and insurance status as a proxy for socioeconomic status are applied, these adjustments did not fully account for the disparity in survival in Hispanic patients, calling for a more comprehensive understanding of contributing factors across this population (7).

Recognizing the critical need for advancing pediatric oncology care in Latin America, the Pediatric Hematology-Oncology Association of Central America (AHOPCA) was formed in 1998, which promoted shared clinical guidelines and uniform approaches to standards of care. Results from AHOPCA-ALL 2008, a Berlin-Frankfurt-Münster (BFM)-based guideline, are well described (11, 12). While countries such as Argentina, Chile, and Uruguay have participated in international, collaborative clinical trials as part of the ALL-IC BFM consortium (13, 14) and Brazil has a long-standing record of national, multicenter randomized controlled clinical trials, however, the broad implementation and/or dissemination of similar multisite, evidence-based, and consensus-derived systematic approaches from countries across the region is lacking (15–18). As a result, in 2018, pediatric cancer centers in 5 countries in South America (Bolivia, Ecuador, Peru, Paraguay, and Venezuela) and St. Jude Children’s Research Hospital (in the USA) formed the Pediatric Oncology of Latin America (POLA), a collaborative group aiming to improve outcomes for children with cancer in the region. Challenges identified by POLA during their 2018-2019 cooperative group meetings included varied access to health services, limited or inconsistent availability of chemotherapies, high abandonment rate, significant diversity in institutional approaches to the risk classification of ALL, and lack of standardized treatment regimens and supportive care guidelines. As a result, a formal retrospective study was planned and conducted.

This paper describes the results from a retrospective review of demographic, biological, and clinical parameters of children under 18 years of age with newly diagnosed ALL presenting between 2013-2017 across five collaborating pediatric centers in four South American countries (Bolivia, Ecuador, Peru, and Paraguay). The interim descriptive and survival analyses have helped to inform the development of an evidence-based, consensus-derived, adapted treatment guideline, which now serves as the new standard of care for pediatric ALL in participating institutions.

## Materials and methods

The Institutional Review Board (IRB) at St. Jude Children’s Research Hospital (SJCRH) reviewed and granted an exemption. The study only required a review of medical records that already existed and did not involve any intervention or treatment of participants therefore a waiver of consent was granted. Additional approval was obtained by all local IRBs or equivalent review processes.

### Study design and participants

This multicenter retrospective cohort study analyzed medical records across five collaborating centers in South America that treat pediatric cancer, including Caja Petrolera Salud (Santa Cruz, Bolivia), Sociedad de Lucha Contra El Cancer SOLCA (Loja, Ecuador), SOLCA (Quito, Ecuador), Hospital de Clínicas Universidad Nacional de Asunción (Asunción, Paraguay), and Instituto Nacional de Enfermedades Neoplásicas (INEN, Lima, Peru). Health records were maintained by participating sites. Participants included children and adolescents less than 18 years old with newly diagnosed B- or T-cell ALL between January 1, 2013, and December 31, 2017. Immunophenotype was determined by morphological evaluation and immunophenotyping by flow cytometry, if available. Patients were risk-classified and treated by each respective institutional treatment regimen. The number of risk groups and the criteria used for initial and final risk classification varied between institutions.

### Data collection

Demographic and clinical data were collected using TrialMaster, a web-based database, and guided by a case report form (CRF) identical to the one used in the MAS study to standardize data collection across the region. Demographic information included age and gender. Clinical information included date of diagnosis, immunophenotype, risk classification, treatment, laboratory tests, molecular characteristics, and follow-up. Local investigators designated a data collection team. Five teams, including eighteen physicians, participated in virtual training, including Collaborative Institutional Training Initiative (CITI) training, data abstraction training through Cure4Kids (cure4kids.org), two 1-hour reviews of the case report form, three 1-hour live sessions to review TrialMaster functionality, address queries, generate reports, and practice entering data from an example patient case into TrialMaster. The CRF was developed in English, translated into Spanish, and reviewed by bilingual members of the research team. A bilingual research team member conducted the data abstraction training courses in Spanish. Data from eligible patients were abstracted from hospital census records, pathology databases, and other local sources and coded directly into the password-secured electronic database. A unique study identifier was used to limit identifiable data collection. Study staff at St. Jude reviewed the data monthly and queried sites for missing information or clarification.

Data collection started in November 2019 and was planned to end in December 2020. However, due to the COVID-19 pandemic, the period for data collection was extended to December 2021. Patients whose complete clinical records were not available or accessible during this time were not included.

### Statistical analysis

Patient characteristics were summarized using descriptive statistics. Continuous data were summarized using means and standard deviations (SD) or medians and interquartile range (IQR), and percentages were used to summarize categorical data. EFS and OS were calculated using the Kaplan-Meier method with standard errors of Peto et al. (19, 20). EFS was defined as the time from diagnosis to first event (induction failure, induction death, relapse, remission death) or date of last contact for those who were event-free. Induction failure was defined as the presence of ≥25% leukemic blasts in the bone marrow after remission induction treatment (end of induction as defined by the institutional treatment regimen). Induction death was defined as death prior to achieving complete remission (CR, <5% leukemic blasts in the marrow) at the end of induction. Marrow relapse was defined as ≥25% blasts in the bone marrow by morphology or flow cytometry-based evaluation. Remission death was defined as death after achieving CR. The OS was defined as the time from diagnosis to death or last contact if still alive. For Abandonment-sensitive EFS (AEFS) and OS (AOS), treatment abandonment was considered an event. Abandonment was defined as a period of >4 weeks without curative treatment, not due to toxicity or other medical causes. Substantial change to therapy was defined as the elimination or substitution of a drug for more than half of the dose of a treatment phase. Log-rank test was used to compare survival curves between groups (21, 22). Cumulative incidence rates were computed using the cumulative incidence function for competing risks, and comparisons between groups were made using the *K*-sample test (23). Univariate and multivariable logistic regression analyses were used to identify factors affecting minimal residual disease (MRD). Factors significant in univariate analyses were included in the multivariable models, and odds ratios and 95% confidence intervals (CIs) were calculated. Univariate and multivariable Cox regression analysis were used to study the effect of factors on EFS. For all analyses, a p-value <0.05 was considered statistically significant. Analyses were done using SAS software, version 9.4, and R version 4.0.0.

## Results

### Demographics and clinical characteristics

During the 5-year study period, 752 children with ALL were diagnosed, and follow-up data were available for 746. Median follow-up time was 3.52 years (IQR 3.07). The demographics and clinical characteristics are summarized in **Table 1**. A total of 54.4% (n=409) were male, with a median age of 5.5 years old (IQR 7.29). B-cell ALL was diagnosed in 92.1% (n=690) of patients and 7.5% (n=56) with T-cell ALL. The median white blood cell (WBC) at diagnosis was 11.7x10^3^/µL (IQR 36.5x10^3^/µL). A mediastinal mass was noted in 4.1% (n=31) of patients at presentation, with 0.2% (n=1) reported to have testicular involvement. Patients with Down syndrome comprised 1.8% (n=13) of ALL cases.

**Table 1:** Characteristics of included patients.

	%, N=752
Treatment center	
Caja Petrolera Salud- Santa Cruz, Bolivia	1.2 (9)
Sociedad de Lucha Contra El Cancer SOLCA- Loja, Ecuador	5.3 (40)
Sociedad de Lucha Contra El Cancer SOLCA-Quito, Ecuador	24.1 (181)
Hospital de Clínicas Universidad Nacional de Asunción-Asunción, Paraguay	26.2 (197)
Instituto Nacional de Enfermedades Neoplásicas- Lima, Peru	43.2 (325)
Sex	
Male	54.4 (409)
Age (years)	
Median, IQR	5.52 (7.29)
Immunophenotype	
B-cell	92.1 (690)
T-cell	7.5 (56)
Mediastinal Mass	4.1 (31)
Testicular involvement	0.2 (1)
Total WBC at diagnosis (x10^3^/µL), Median, (IQR)	11.7, (36.5)
Risk group at the start of treatment	
Standard	47.8 (357)
High	51.9 (388)
CNS status at diagnosis	57.3 (429)
CNS 1 (0 blasts)	93.7 (402)
CNS 2 (>1 blasts, <5 leukocytes/HPF)	1.6 (7)
CNS 3 (>1 blasts, >5 leukocytes/HPF)	0.7 (3)
Cranial Nerve Palsy	1.9 (8)
Results not available	2.1 (9)
Not evaluated	42.7 (320)
Risk group at end of induction	
Standard	36.5 (275)
High	50.0 (375)
Very high	3.9 (29)
Results not available	2.1 (16)
Missing	7.6 (57)
Follow-up information available	99.2 (746)

### Diagnostic assessment

Central nervous system involvement was only evaluated at diagnosis in 57.3% (n=429) of patients; of this subset, 93.7% (n=402) patients were CNS 1, 1.6% (n=7) were CNS 2, 0.7% (n=3) were CNS 3, 1.9% (n=8) had a documented cranial nerve palsy, and 2.1% (n=9) results were not available. A traumatic tap (>10 RBC/µL) was observed in 19 patients; of these 16 were categorized as CNS1 and 3 as CNS2.

The median time from diagnosis to the start of treatment was 4 days (range: 1-34), and the time from presentation to advanced result confirmation for cytogenetics, fluorescent *in situ* hybridization (FISH), and polymerase chain reaction (PCR) testing was 13 days, 11 days, and 15 days respectively. Across centers, only 23.4% (n=175) of patients received FISH results, and 56.5% (n= 422) received PCR testing. The frequency of advanced testing varied by treatment location. During the study period, cytogenetics was routinely obtained in 2 centers, FISH in 1 center, and PCR in three centers. Testing for t (9, 22) BCR-ABL1 fusion was the only advanced diagnostic test uniformly obtained across all five treatment centers and positive in 4.4% (n=24/550) (**Table 2**). Of patients with results available, low-risk features ETV6-RUX1 and hyperdiploidy (>50) were observed in 14.1% (n=76/538) and 15.5% (n=53/341) of patients. Traditionally high-risk (HR) features, TCF3-PBX1, iAMP21, and KMT2A-rearranged ALL, were observed in 8.3% (n=43/521), 1.1% (n=11/99), and 2.2% (n=11/503) of patients respectively (**Table 2**).

**Table 2:** Treatment outcomes according to clinical and biological characteristics.

Characteristic	No. of Patients	3-Year EFS% (± SE)	*P*	3-Year OS% (± SE)	*P*
All patients					
*Overall Abandonment sensitive*	746	60.96 ± 1.86	–	67.19 ± 1.80	–
*Overall*	746	71.02 ± 1.85	–	79.59 ± 1.67	–
Age					
<1 year	14	50.00 ± 13.36	<0.0001	50.00 ± 13.36	<0.0001
1-10 years	525	75.84 ± 2.07		83.98 ± 1.80	
>=10 years	207	60.21 ± 3.84		70.57 ± 3.65	
*Trisomy 21*					
* Yes*	13	53.85 ± 14.93	0.1078	68.38 ± 14.53	0.1445
* No*	724	71.46 ± 1.86		79.86 ± 1.68	
Ploidy					
>50 chromosomes	53	83.35 ± 5.92	0.1829	89.52 ± 4.90	0.1813
≤50 chromosomes	285	75.82 ± 2.83		81.10 ± 2.62	
Translocations					
t(9;22)(BCR-ABL1)	24	47.73 ± 11.50	<0.0001	60.67 ± 12.03	<0.0001
t(12;21)(ETV6-RUNX1)	76	85.73 ± 4.29		91.55 ± 3.41	
t(1;19)(TCF3-PBX1)	43	81.08 ± 6.79		86.29 ± 6.15	
KMT2A-rearranged ALL	11	N/A		11.67 ± 10.97	
iAMP21	11	81.82 ± 14.24		81.82 ± 14.24	
Treatment modification					
Substantial change	72	51.42 ± 6.54	0.0028	65.56 ± 6.50	0.0219
No substantial change	673	73.24 ± 1.90		81.12 ± 1.70	
B-cell ALL					
*All B-cell*	687	72.76 ± 1.89	–	80.70 ± 1.70	–
CNS status					
CNS-1	352	73.79 ± 2.54	0.0970	78.96 ± 2.36	0.0598
CNS-2/CNS-3	7	33.33 ± 19.25		50.00 ± 20.41	
Traumatic	18	61.11 ± 11.49		71.43 ± 11.02	
WBC					
<20,000/µL	442	79.21 ± 2.13	<0.0001	86.00 ± 1.84	0.0007
20-50,000/µL	114	64.76 ± 5.09		71.10 ± 4.85	
50-100,000/µL	62	55.84 ± 6.89		69.41 ± 6.68	
≥100,000/µL	64	56.15 ± 7.29		69.90 ± 7.25	
Initial risk group					
Standard risk	353	79.61 ± 2.31	0.0001	86.14 ± 2.00	0.0148
High risk	332	65.23 ± 3.02		74.71 ± 2.82	
MRD Day 15					
Negative	72	80.08 ± 4.91	0.5961	80.06 ± 4.91	0.1970
Positive	43	81.40 ± 7.17		88.25 ± 5.93	
MRD Day 29					
Negative	74	95.32 ± 2.78	<0.0001	96.88 ± 2.29	0.0002
Positive	24	51.15 ± 11.31		84.69 ± 9.19	
T-cell ALL					
All T-cell	56	49.85 ± 7.21	–	65.95 ± 7.15	–
CNS status					
CNS-1	28	50.00 ± 9.45		59.86 ± 9.48	

“-“ No relevant comparison.

NA, not applicable.

At the start of treatment, 51.9% (n=388) of patients were categorized as high-risk based on the site-specific treatment regimens. Heterogeneity was observed among risk classifications with variations in naming, factors considered, and the number of risk groups ranging from 2-4 final groups across the different centers. The risk factors considered included age, WBC at diagnosis, presence of favorable or unfavorable biologic features, immunophenotype, end of induction response, and prednisone response in BFM-based regimens. Final risk classification was determined based on each center’s treatment regimen and incorporated disease status at the end of induction.

### Treatment regimens

Patients were treated based on the institutional pediatric ALL regimen which varied across all 5 sites. Patients were treated according to an unmodified St. Jude Total XV regimen at SOLCA Quito and the ALL-Intercontinental-BFM/PINDA (National Chilean Pediatric Oncology Group) 2009 regimen in Caja Petrolera Salud in Bolivia. At the remaining 3 centers, patients were treated according to a locally derived regimen (INEN Protocolo LLA 2014, LLA Paraguay Protocolo 2008, LLA Solca 2012). Of the 3 locally derived regimens, 2 were based on a BFM treatment backbone. Regimen modifications were ascertained to understand capacity-informed changes at the local level. Examples of local modifications included (1) delaying the first intrathecal (IT) until day 5-7 of induction, (2) mid-study change in intrathecal therapy due to drug availability, and (3) reducing the dose of methotrexate in consolidation from 5g/m^2^ to 2gm/m^2^ over 4 hours for patients with B-cell ALL. Reduced methotrexate was given to facilitate outpatient administration to accommodate limited inpatient bed availability and minimize treatment delays.

Prior to confirmatory diagnosis, 5% (n=37) of patients had received pretreatment. Most prior treatments consisted of steroids alone 62.1% (n=23), or a combination of steroids, systemic chemotherapy, or intrathecal therapy. Treatment regimens from 3 of the 5 sites included a 7-day steroid prephase. Across the other two sites, an additional 13.8% (n=70/506) of patients received steroids before combined systemic therapy. The steroid of choice during prephase was prednisone in 65.0% (n=210), dexamethasone in 34.1% (n=110), and methylprednisolone in 1.5% (n=5) of patients.

During induction therapy, most patients received a four-drug induction with steroids, vincristine, L-asparaginase, and anthracyclines. Due to the dosing heterogeneity and drug substitutions between the different regimens, the average doses were computed to inform future harmonization efforts. These included mean prednisone equivalents 1,571 mg/m^2^ (IQR 723mg/m^2^) and cumulative median doxorubicin equivalent dose 75 mg/m^2^ (IQR 70mg/m^2^). Most patients received daunorubicin 66.2% (n=482) compared to doxorubicin 33.8% (n=242); 10 patients received both. Only native E. coli L-asparaginase was available for asparagine depletion; no patients at any sites received pegylated (PEG) L-asparaginase during the observation period. The planned number of doses of asparaginase during induction varied across different treatment regimens with 2 regimens using 5,000U/m^2^ x 8 doses and the other 3 regimens using 10,000U/m^2^ x 6 doses.

For CNS-directed therapy, patients received a median of 5 doses (IQR 4 doses) of IT chemotherapy during induction. The IT therapy varied by location. In Paraguay, both single-agent IT dexamethasone and triple IT (methotrexate, cytarabine, and dexamethasone) were used. A triple IT was also utilized in Ecuador composed of methotrexate, cytarabine, and hydrocortisone. In Bolivia, single-agent IT methotrexate and IT hydrocortisone were used. At the start of the study, Peru used triple IT with methotrexate, cytarabine, and dexamethasone, however, in 2015, they transitioned to “double” IT with methotrexate and cytarabine due to a lack of drug formulation availability. Radiation therapy was administered to 3.5% (n=24) of patients (n=6 cranial, n=18 craniospinal) with a median dose of 1800Gy (range 1000-3000Gy).

### Chemotherapy response

At the end of induction (Day 29), 84.8% (n=588) of patients had an M1 marrow by morphology, 9.1% (n=63) had an M2 marrow, 1.9% (n=13) had induction failure with an M3 marrow, and data was not available in 4.2% (n=29). Minimal residual disease (MRD) was evaluated in 69.1% (n=516) of patients. Of the patients with end-of-induction MRD as per their institutional regimen (Day 29, Day 42, or Other), 70.4% (n=207/294) were MRD negative (<0.01%) (**Table 3**).

**Table 3:** MRD monitoring for B-cell ALL.

Characteristic	Value (%)
Evaluation of MRD, n (%)	
Yes	516 (69.1)
No	231 (30.9)
MRD D8, n (%)	91 (17.6)
<0.01%	40 (44.0)
0.01-0.99%	28 (30.8)
>=1%	23 (25.3)
MRD D15, n (%)	131 (25.4)
<0.01%	82 (62.6)
0.01-0.99%	26 (19.8)
≥1%	22(16.8)
Not sent/not interpretable	1 (0.8)
MRD D29, n (%)	112 (21.7)
<0.01%	82 (73.2)
0.01-0.99%	21 (18.8)
≥1%	8 (7.1)
Not sent/not interpretable	1 (0.9)
MRD D42, n(%)	35 (6.8)
<0.01%	19 (54.3)
0.01-0.99%	9 (25.7)
≥1%	6 (17.1)
Not sent/not interpretable	1 (2.9)
MRD other, n(%)	147 (28.5)
<0.01%	106 (72.1)
0.01-0.99%	24 (16.3)
≥1%	16 (10.9)

Following completion of the induction block, risk classification was changed in 15.3% (n=106) of patients. These changes were attributed to steroid pretreatment in 1.9% (n=2), biological features including FISH/cytogenetic results and DNA index in 6.6% (n=7), poor treatment response in induction in 88.7% (n=94), and not categorized/other in 2.8% (n=3) of patients. Minimal residual disease was used to determine treatment response in 60.4% (n=64). The resulting final risk group composition included 39.6% (n= 275) Standard Risk (SR), 54.0% (n= 375) High Risk (HR), and 4.2% (n=29) Very High Risk (VHR) per institutional risk classification system. Final risk classification was not available in 2.3% (n=16) of patients.

**Figure 1:**
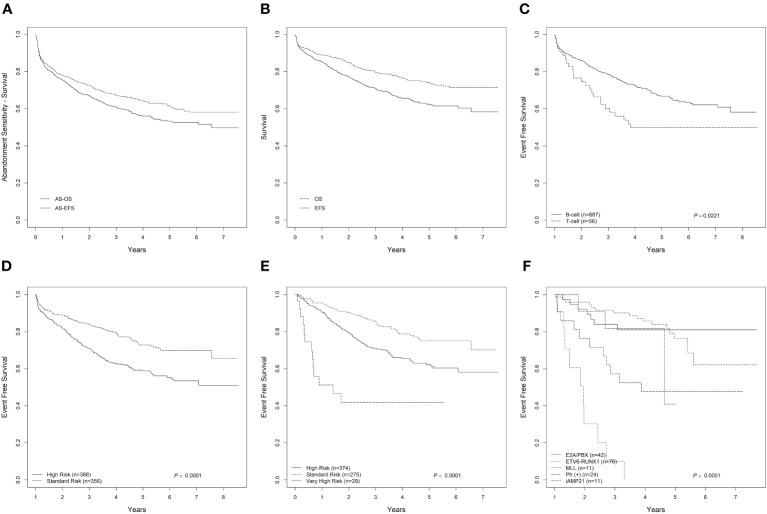
Outcome of pediatric ALL in 4 countries in South America. **(A)** Abandonment-sensitive EFS and OS for ALL; **(B)** Overall EFS and OS for ALL; **(C)** EFS by for B-cell and T-cell ALL; **(D)** EFS by Initial Risk Group classification based on institutional treatment regimen; **(E)** EFS by Final Risk Group classification based on institutional treatment regimen; **(F)** EFS by molecular biology for ALL.

The AEFS and AOS were 61.0 ± 1.9% and 67.2± 1.8% at 3 years and 53.3± 3.2% and 61.5± 3.1% at 5 years, respectively (**Figure 1A**). The overall EFS and OS were 71.0 ± 1.8% and 79.6 ± 1.7% at 3 years and 62.5 ± 3.3% and 74.4 ± 3.0% at 5 years, respectively (**Figure 1B**). Due to small patient numbers and insufficient follow-up among those, only 3-year survival rates are reported for the various subgroups analyzed. Patients with B-cell ALL had a higher 3-year EFS at 72.8 ± 1.9% compared to T-cell ALL at 49.9 ± 7.2% (EFS p=0.0221) (**Figure 1C**). Based on initial risk classification, the 3-year EFS and OS for SR were 79.5 ± 2.3% and 85.9 ± 2.0% compared to 62.9± 2.8% and 73.5 ± 2.6% for HR (EFS p<0.0001; OS p=0.0076) (**Figure 1D**). Evaluation of survival outcomes based on final risk groups revealed a 3-year EFS for SR of 85.6 ± 2.3%, HR 70.9 ± 2.6%, and VHR 41.9 ± 11.3%, and 3-year OS for SR of 92.9 ± 1.7%, HR 81.0 ± 2.3, and VHR 55.8 ± 13.1% (EFS p<0.0001; OS p=0.0001) (**Figure 1E**). Analyses of AEFS and AOS by immunophenotype and risk groups are provided in the supplement (**Supplemental Figure 1**).

For patients with B-ALL who were MRD negative (<0.01%) on day 29, the 3-year EFS was 95.3 ± 2.8% (p<0.001), and the OS was 96.9 ± 2.3 (p<0.001). The higher the level of MRD positivity at the end of induction, the lower the 3-year EFS with MRD 0.01-0.99% survival decreased to 57.9 ± 13.3%, and MRD ≥1% survival decreased to 33.3 ± 19.3% (p<0.0001).

This study also evaluated the impact of missed asparaginase. Incomplete asparaginase dosing, defined as missing one or more planned doses, was observed in 183 patients. These patients received a median of 50.9% (IQR 38.7) of the planned asparaginase doses for their treatment regimen and this group had a reduced 3-year EFS and OS at 51.2 ± 5.3% and 60.9 ± 5.3% compared to an EFS of 75.7 ± 1.9% and OS 84.2 ± 1.7% for patients who received an entire course (p<0.0001). This finding, however, is likely multifactorial given other aspects of treatment which could have been affected.

### Impact by presenting features

Age and WBC at presentation were associated with poorer EFS and OS. Patients with age (≥1 and <10 years old) had a 3-year EFS of 75.8 ± 2.1% compared to children < 1-year-old and ≥ 10 years old (50.0 ± 13.4% and 60.2 ± 3.1%, respectively). Children with WBC < 20x10^3^/µL at presentation had a 3-year EFS of 79.2 ± 2.13% compared to 64.8 ± 5.09% for patients with WBC 20-50x10^3^/µL (p<0.0001). The 3-year EFS for patients with an initial WBC ≥100x10^3^/µL (56.2 ± 7.29%) was no worse than for patients with an initial presentation of 50-100x10^3^/µL (55.8 ± 6.89%) (**Table 2**). In multivariable Cox regression analysis, the difference in outcome by age remained significant after adjusting for day 29 MRD (HR 1.18, p=0.0203) (**Supplemental Table 1**). Univariate logistic regression showed that low WBC at diagnosis and lower final risk group classification were associated with negative MRD status on Day 29 (p=0.014 and p=0.0031, respectively) (**Supplemental Table 2**).

Survival significantly varied according to molecular characteristics at diagnosis (**Table 2**, **Figure 1F**). Patients with ETV6-RUNX1 (n=76) had a 3-year EFS and OS 85.7 ± 4.29% and 91.5 ± 3.41% respectively (EFS p<0.0001, OS p<0.0001). For the 24 patients with BCR-ABL1, the 3-year EFS and OS were 47.7 ± 11.50% and 60.7 ± 12.03%, respectively. Notably, while 24 patients were positive for BCR-ABL1 fusion, only 50% (n=12) received a tyrosine kinase inhibitor (imatinib) as part of the systemic therapy.

### Toxicity and treatment-related morbidity

During the induction phase, 84.7% (n=631) of patients received a therapeutic course of antibiotics. Commonly observed infections included clinical sepsis in 55.3% (n=184), pneumonia in 49.8% (n=166), bacteremia in 44.1% (n=147), and meningitis in 0.6% (n=2) patients.

Substantial changes to the original treatment regimen were observed in 9.6% (n=72) of patients, with the most common causes including infection in 30.6% (n=22), modification at the start of treatment due to serious illness or clinical condition in 8.4% (n=6), and drug availability in 5.6% (n=4) of patients. Treatment delays of 2 weeks or more were observed in 56.0% (n=421) of patients (median duration 15 days). The most common causes of treatment delay included infection 63.2% (n=265) and other toxicity 47.3% (n=198). Varicella zoster infections were observed in 6.2% (n=46), resulting in a median 13-day (IQR 10) delay in therapy. Interestingly, patients who required treatment delay >2 weeks had a higher EFS (75.1 ± 2.3%) compared to the patients who did not experience treatment interruptions (66.0 ± 3.1%) (p=0.0002), however considering those who completed induction and continued to post-induction therapy the 3-year OS was not statistically significant between the two groups (p= 0.6352). Comparing events between the two groups, in the patients with treatment held > 2 weeks (n=421), relapse and death were observed in 95 and 20 patients, respectively, versus 52 and 61 patients in the group without treatment interruptions (n=325). The 3-year OS was 83.8 ± 2.0% for those with a treatment delay versus 74.1 ± 0.29% for those with no prolonged delay (p<0.0001).

Asparaginase-specific toxicities were evaluated (**Table 4**). Asparaginase-associated toxicity resulting in dose omission was observed in 18.9% (n=141) of patients. A third of patients (n=47) only required intermittent dose omission, while 2/3 (n=94) necessitated permanent discontinuation due to severe toxicity or lack of access. The most common reasons for dose omission or discontinuation of asparaginase included: systemic hypersensitivity 22% (n=31), infection 14.2% (n=20), local reaction 10.6% (n=15), pancreatitis (mild in 5.7% [n=8]; severe in 4.3% [n=6]), thrombosis 1.4% (n=2), and drug availability 2.1% (n=3).

**Table 4:** Asparaginase associated toxicities.

	N=727 (%)
Asparaginase	
Systemic Hypersensitivity	39 (5.4)
Local reaction	27 (3.7)
Hypofibrinogenemia	16 (2.2)
Pancreatitis	19 (2.6)
Mild	11 (1.5)
Severe	8 (1.1)
Hyperglycemia	9 (1.2)
Thrombosis	7 (1.0)
Indirect hyperbilirubinemia	1 (0.1)

While most patients received all four doses of planned high-dose (HD)-methotrexate, 6.5% (n=44) of patients experienced a toxicity or access issue requiring dose omission. Of these patients, 84.1% (n=37) required permanent discontinuation due to severe toxicity or lack of access. The most common reasons for dose omission or discontinuation of methotrexate included: abandonment 67.6% (n=25), severe mucositis (> Grade 3) 9.1% (n=4), transfer to a different treatment center 8.1% (n=3), encephalopathy 6.8% (n=3), infection 4.5% (n=2), drug availability 4.5% (n=2), and vision loss 2.7% (n=1). Five patients discontinued HD-methotrexate due to relapse.

At the time of analysis, 13% (n=97) of patients had abandoned treatment, and 1.9% (n=14) had transferred to other treatment facilities.

### Treatment-related mortality

During the study period, 19.5% (n=164) of patients died, with early deaths represented by an induction death rate of 6.4% (n=48/746) and a remission death rate of 3.8% (n=29/746). Infection was the most common cause of death during induction 89.6% (n=43). Of remission deaths, 20 occurred while the patients were still on therapy, of these 80% (n=16) were due to toxicity, and 9 occurred off therapy. Nine deaths were recorded following treatment abandonment or transfer to an alternate treatment center.

### Relapses

Relapse was observed in 19.8% (n=147) of patients (isolated bone marrow n=84, isolated CNS n=35, mixed relapse n=23 [marrow and CNS], non-CNS extramedullary n=5). The cumulative incidence of isolated bone marrow relapse was 9.52 ± 1.14%, isolated CNS relapse was 4.35 ± 0.79%, and mixed relapse was 2.42 ± 0.59% at 3 years. There was no significant difference in the cumulative incidence of bone marrow (p=0.5165), isolated CNS (p=0.4850), or mixed relapse (p=0.3378) between those who received complete (n=599) vs incomplete asparaginase dosing (n=138).

## Discussion

The survival outcomes from this study align with recently published estimates of pediatric ALL survival in the region arising from population-based cancer registries and simulation models.(4) These findings are also comparable to pediatric survival outcomes from countries with similar characteristics such as Brazil, Columbia, Guatemala, and Mexico, with reported 5-year OS of 52-69.5%, 55.6%, 64.1%, 61.8%, respectively, as well as regional collaborative groups such as AHOPCA with a 3-year AOS of 68.2% (12, 16, 24–27). However, outcomes remain lower than those achieved by Argentina, Uruguay, and Chile with 5-year OS of 86.2% as part of the international cooperative trial, ALL-IC-BFM 2009 (14). These results demonstrate a persistent survival gap compared with outcomes achieved through modern ALL regimens in higher-income settings (1–3, 7). While the historical emphasis on understanding gaps in survival has focused on either leukemia biology or local capacity, this evaluation attempts to bring together these multifactorial challenges and inform the development of regional initiatives. To support the goal of POLA in advancing cures for children with ALL these two elements, biology vs. capacity can be reframed as fixed or modifiable factors that can guide appropriate strategies to address current challenges and direct future efforts.

Fixed factors represent patient and clinical characteristics unique to pediatric ALL in the region, such as age, gender, immunophenotype, and unique leukemic profile. In this population from South America, a higher proportion of high-risk genetic markers and a lower proportion of low-risk molecular markers have been observed compared to the results from children in two collaborative groups in North America (7, 28, 29) While the observed pattern in this study is similar to results from a single center in Guatemala, the population in South America had a uniquely lower frequency of hyperdiploidy (15.6% vs. 24.1%) (26). This highlights a need to better characterize and understand the biological characteristics defining ALL across these diverse geographic and ethnic regions and given the potential for high-risk features, the need to proactively incorporate risk stratification and modern, targeted, and supportive care therapies to improve survival. Blind increases in treatment intensity without investment in comprehensive modern risk stratification can result in a detrimental influence on survival secondary to treatment-related morbidity and mortality. This retrospective study has provided insight into the frequency of these specific biological variations. Still, prospective work is needed to comprehensively evaluate prognostic features and measure the impact of context-appropriate, risk-adapted treatment elements on survival.

Applying an implementation lens to a well-described problem, this study also demonstrated capacity-based challenges or modifiable factors. These factors span multiple levels of the health system, including limitations in drug access to essential cancer medicines at the national level, supportive care and laboratory capacity at the hospital level, and socioeconomic challenges which contribute to persistently high rates of abandonment (13%) and treatment interruption at the patient level.

Across institutions, significant treatment-related toxicity was observed, including a high frequency of infections and drug-related toxicity. These infections resulted in substantial treatment delays and contributed to further resource strain in institutions where infrastructure limitations already impede care delivery. For example, providers indicated that 97 patients had treatment held because of resource constraints including bed availability. Treatment delays and drug omissions were also attributed to drug availability. There is a critical need to address the infrastructure for essential medicines like L-Asparaginase, but also proactively combat delayed translation of newer therapies replacing these drugs as the standard of care evolves, such as PEG-asparaginase and recombinant asparaginase. This rationale should also extend to targeted treatments such as tyrosine kinase inhibitors and bi-specific antibodies, which need to be investigated and may have a more significant relative benefit for populations with difficult-to-treat leukemia. The lack of access to cancer-directed therapy highlights the need for improved access to high-quality drugs and opportunities for innovative mechanisms such as the Global Drug Access Platform (30). This cross-sector collaborative effort with the World Health Organization, St. Jude Children’s Research Hospital, and global partners will forecast medication needs and work with local governments to increase access to an uninterrupted supply of essential cancer medicines.

Extending the theme of access challenges, this study demonstrated a clear need for improved laboratory capacity to support each step of clinical management. The feasibility and availability of advanced testing were widely varied across centers with cytogenetics, ETV6-RUNX1, BCR-ABL1, TCF3-PBX1, KMT2A-rearranged ALL, iAMP21, and DNA index accessible in 1.6-73% of cases (**Table 5**), and MRD available for 69.1% of patients. The disproportionately low proportion of patients with CNS involvement also highlights potential diagnostic challenges contributing to misdiagnosis. First, many patients lacked intrathecal evaluation prior to starting therapy due to clinical condition or provider comfort which may have resulted in missed diagnoses of CNS involvement. Second, the differentiation of CNS 2 required significant laboratory skill and expertise which may have compounded the underdiagnosis of CNS involvement. At the patient level, the limited feasibility of advanced diagnostic testing and MRD-based treatment response is a critical barrier to risk stratification and treatment intensification necessary to improve cures. More broadly, the lack of complete biological and genetic data about leukemic subtypes in these patients dramatically limits the ability to identify relevant risk factors for this growing population and inform adapted treatment to further improve survival outcomes in Hispanic children across this geographic and ethnically diverse region. This problem is not limited to patients living in this region but extends to the needs of patients with a shared background worldwide.

**Table 5:** Availability of advanced testing and biological characteristics of 752 patients who participated in the study, 3 data points were missing.

	Advanced Testing Sent n(%), N=749	Results Available n(%), N=749
Karyotype	472 (63)	341 (46)
DNA Index	12 (2)	2 (1.6)
t(12;21) ETV6-RUNX1	592 (79)	538 (72)
t(1;19) TCF3/PBX1	577 (77)	521 (70)
t(9;22) BCR-ABL1	604 (81)	550 (73)
t(4;11) KMT2A-rearranged ALL	558 (75)	503 (67)
iAMP21	316 (42)	101 (13)
*Range*	*2-81%*	*1.6-73%*

Of the remaining 749 patients, advanced testing was sent for 2-81% of patients, depending on the test. Results available are represented by the sum of all resulted tests (positive and negative), excluding results listed as “not available” or “not interpretable.”.

There was also heterogeneity in treatment regimens utilized between the different real-world centers. Not only did institutions rely on different treatment backbones, but there was also variation in risk classification systems, the role and duration of steroid prephase, local availability of systemic and intrathecal chemotherapy, practices for CNS evaluation (before or after prephase), duration of induction (29 versus 42 days), and disease evaluation endpoints as demonstrated by the range of time points for MRD based disease evaluation on Day 8, 15, 29, or 42. The various time points for MRD-based disease evaluation prevented a robust comparison of early treatment response between the different regimens. Appreciating the current differences and understanding the rationale of approaches observed from this multinational study was essential to guide the next steps of collaborative expansion.

## Limitations

While this study was multinational and conducted across varied contexts, as a retrospective study, the evaluation was limited by a short follow up time (median follow up 3.52 years) and missing or incomplete data. This was exacerbated by the onset of the COVID-19 pandemic when pediatric oncology resources were shifted to combat the increased stress of the global health system (31). This created an additional burden on practicing clinicians who assumed the role of data collection and database entry and further limited access to complete medical records.

## Conclusion: informing future collaborative efforts

This evaluation demonstrates the multifactorial contributions to poorer survival for children in South America, including non-modifiable variations in ALL biological characteristics and opportunities to improve laboratory capacity, drug access, and supportive care in the region. This collaborative effort requires a conscious balance of strategies to address these fixed and modifiable challenges to enhance the outcomes of children with ALL in the region. Since the completion of this evaluation, additional regional enthusiasm has grown, and these considerations have guided the development of a feasible, evidence-based, consensus-derived, multinational treatment guideline with prospective data collection for pediatric ALL in the South American region, POLA (Pediatric Oncology Latin America) LLA00, which began enrollment in June 2023. Based on the experience with the retrospective evaluation, the POLA regimen includes specific resource-adapted guidelines for risk assignment, common diagnostic and treatment approaches, option for high-dose methotrexate administration based on resources, treatment modifications during drug shortages, dose adjustments to organ function and drug interactions, and an (Adapted Resource and Implementation Application) ARIA-guided simplified Adverse Events capturing. Most importantly a prospective registry for data collection was established and data managers at participating centers were trained in data entry. Weekly teleconferences are held to review the cases and discuss any challenges with the collaborating centers. Registry data will be reviewed and validated in real time to ensure data quality. The POLA group has approached the Ministry of Health (MOH) in their respective countries to advocate for this regional initiative. The plan is to maintain the dialog between the various collaborators (MOH, St. Jude Global, and the World Health Organization) to share data from the POLA registry to maintain engagement and inform healthcare systems in the region.

## Data availability statement

The datasets presented in this article are not readily available because In consideration of participant and institutional privacy, requests for data will be considered on an individual basis. If agreed upon by all participating sites, the deidentified datasets will be shared with the requestor. Datasets will be limited to data elements informing the publication. Requests to access the datasets should be directed to Caitlyn Duffy, caitlyn.duffy@stjude.org.

## Ethics statement

The studies involving humans were approved by St. Jude Children’s Research Hospital. The studies were conducted in accordance with the local legislation and institutional requirements. Written informed consent for participation was not required from the participants or the participants’ legal guardians/next of kin in accordance with the national legislation and institutional requirements.

## Author contributions

CD: Writing – original draft, Writing – review & editing. DG: Conceptualization, Writing – review & editing. AL: Conceptualization, Data curation, Writing – review & editing. AKC: Conceptualization, Project administration, Writing – review & editing. GJ: Formal Analysis, Writing – review & editing. YC: Formal Analysis, Writing – review & editing. MD: Formal Analysis, Writing – review & editing. SL: Conceptualization, Data curation, Writing – review & editing. SB: Conceptualization, Data curation, Writing – review & editing. PF: Conceptualization, Data curation, Writing – review & editing. LT: Data curation, Writing – review & editing. EB: Data curation, Writing – review & editing. SJ: Conceptualization, Data curation, Writing – review & editing. MZ: Data curation, Writing – review & editing. RN: Data curation, Writing – review & editing. AS: Conceptualization, Data curation, Writing – review & editing. GS: Conceptualization, Data curation, Writing – review & editing. EV: Data curation, Writing – review & editing. MM: Conceptualization, Writing – review & editing. PF: Writing – review & editing, Conceptualization. SJ: Writing – review & editing, Conceptualization.
