# Supplementary material for: Retrospective analysis of outcomes for pediatric acute lymphoblastic leukemia in South American centers
Source: Front Oncol. 2023 Oct 30;13:1254233. doi: 10.3389/fonc.2023.1254233 (PMC10643117; doi:10.3389/fonc.2023.1254233)
Supplement: Supplementary file 1 [file DataSheet_1.pdf]

Supplementary Material

Retrospective Analysis of Outcomes for Pediatric Acute Lymphoblastic Leukemia in South American Centers

Caitlyn Duffy[1*], Dylan Graetz[1], Arturo M. Zapata Lopez[2], Angela K. Carrillo[1], Godwin Job[1], Yichen Chen[1], Meenakshi Devidas[1], Sandra Alarcón Leon[2], Sol Aponte Bonzi[3], Pedro Cardona Flores[4], Lizeth Escobar Torres[5], Eddy Hernández Broncano[2], Soledad Jiménez Jaramillo[6], Ma Ofelia Zelada[3], Romulo Reaño Novoa[2], Angelica Samudio[4], Gissela Sánchez-Fernandez[5], Erika Villanueva[5], Monika Metzger[7], Paola Friedrich[1†], Sima Jeha[1†]

*** Correspondence:** Caitlyn Duffy: Caitlyn.duffy@stjude.org

1 Supplementary Tables and Figure

Supplemental Table 1: Univariate and multivariable Cox regression analysis of EFS (limited to cohort with Day 29 MRD data)

Effect	Univariate Analysis			Multivariable Analysis (N=109)	
	N	Hazard Ratio	P-Value	Hazard Ratio	P-Value
**WBC (x10^3/mm^3)**			0.0055		0.7585
<50	78	1.00 (Ref)		1.00 (Ref)	
>=50	31	2.89 (1.32 - 6.35)		1.19 (0.40-3.52)	
**Initial Risk Group**			0.1423		
Standard	43	1.00 (Ref)			
High	68	1.90 (0.79 - 4.56)			
**Final Risk Group**			<.0001		0.0002
Standard	38	1.00 (Ref)		1.00 (Ref)	
High	64	1.42 (0.54 - 3.75)		1.08(0.37-3.17)	
Very High	9	20.94 (6.47 - 67.73)		17.51 (3.56-86.19)	
**Pre-treatment**			0.3292		
Yes	3	0			
No	108	1.00 (Ref)			
**Immunophenotype**			0.0278		0.6036
B cells	99	1.00 (Ref)		1.00 (Ref)	
T cells	12	2.87 (1.07 - 7.67)		1.37 (0.42-4.50)	
**Molecular Biology**			0.9372		
Favorable	14	1.00 (Ref)			
Unfavorable	14	0.76 (0.17 - 3.40)			
Neutral	61	0.88 (0.29 - 2.71)			
**Age**			0.1318		
1-10	83	1.00 (Ref)			
>=10	28	1.86 (0.82 - 4.20)			
**Sex**			0.7586		
Male	54	0.88 (0.40 - 1.94)			
Female	57	1.00 (Ref)			
**MRD Day 29 Status**			<0.0001		<0.0001

Positive	29	9.42 (3.91 - 22.72)		9.73 (3.80-24.93)	
Negative	81	1.00 (Ref)		1.00 (Ref)	

Supplement Table 2: Univariate logistic regression analysis of MRD Day 29:

Effect	P-Value	Odds Ratio
WBC (x10^3/mm^3)	0.0144	
<50		1.00 (Ref)
>=50		3.03 (1.22 - 7.53)
Initial Risk Group	0.0604	
Standard		1.00 (Ref)
High		2.46 (0.95 - 6.40)
Final Risk Group	0.0031	
Standard		1.00 (Ref)
High		5.41 (1.49 - 19.65)
Very High		14.17 (2.42 - 82.92)
Pretreatment	0.7733	
Yes		1.00 (Ref)
No		0.70 (0.06 - 8.02)
Immunophenotype	0.1944	
B cells		0.45 (0.13 - 1.54)
T cells		1.00 (Ref)
Molecular Biology	0.5418	
Favorable		1.00 (Ref)
Unfavorable		1.64 (0.23 - 11.70)
Neutral		2.32 (0.47 - 11.46)
Age	0.0668	
1-10		1.00 (Ref)
>=10		2.34 (0.93 - 5.87)

Supplementary Figure 1:

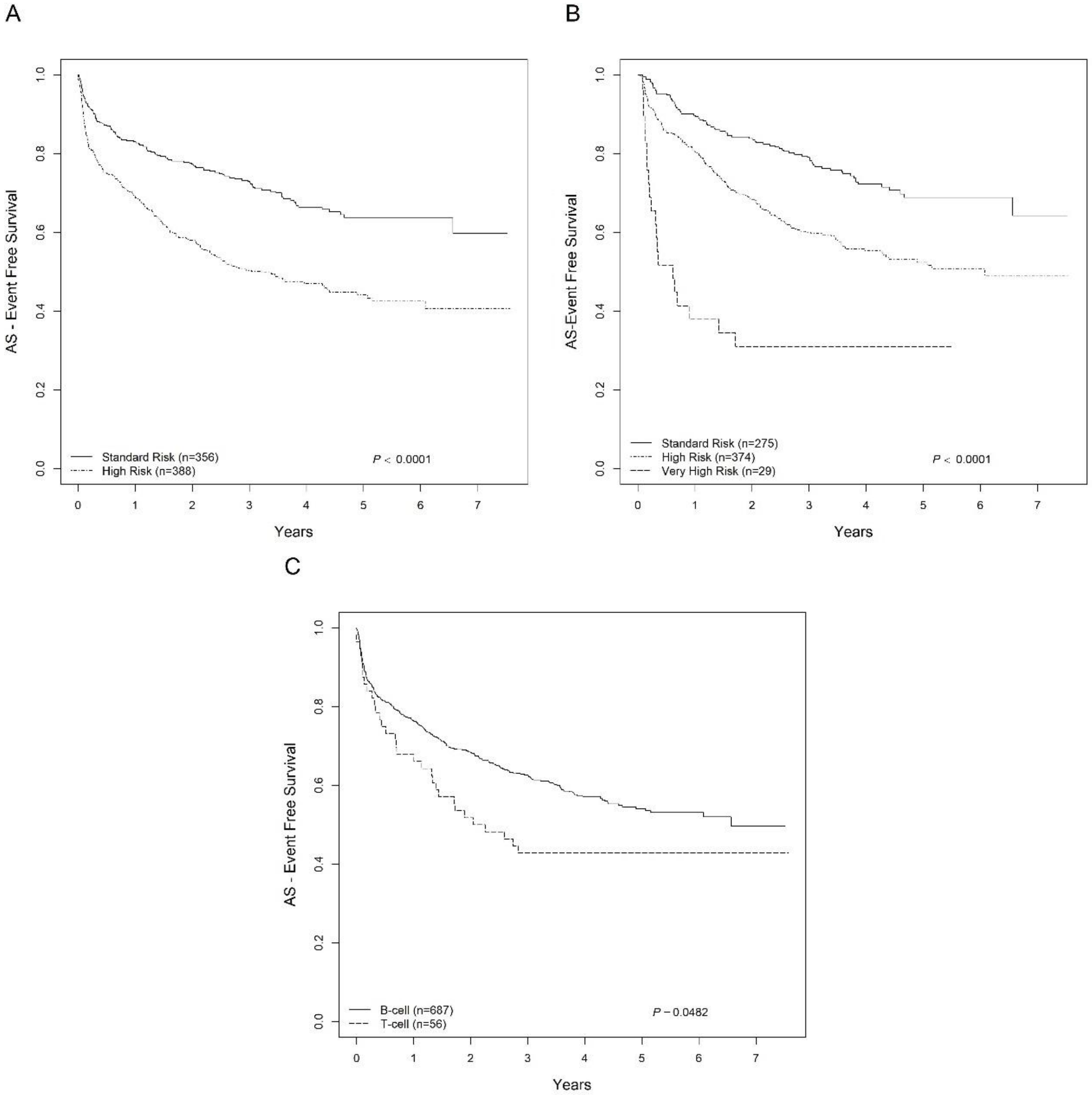

Outcome of pediatric ALL in 4 countries in South America. A) Abandonment-sensitive EFS by Initial Risk Group classification based on institutional treatment regimen; B) Abandonment-sensitive EFS by Final Risk Group classification based on institutional treatment regimen; C) Abandonment-sensitive EFS by Immunophenotype.

Patients with B-cell ALL had a higher 3-year AEFS at 62.6±1.8% compared to T-cell ALL at 42.9±6.6% (AEFS p=0.0482). Based on initial risk classification, 3-year AEFS and AOS for SR were 73.0±1.4% and 78.3±2.3% compared to 50.4±2.7% and 57.4±2.7% for HR (AEFS p<0.0001; AOS p<0.0001). Evaluation of survival outcomes based on final risk groups revealed a 3-year AEFS for SR 79.1±2.5%, HR 60.1±2.7%, and VHR 31.0±9.1%, and 3-year AOS for SR 84.9±2.2%, HR 67.9±2.6%, and VHR 31.1±9.6% (AEFS p<0.0001; AOS p<0.0001).
